# 2372: Metabolite and biomarker predictors of WTC-lung injury: An integrated multiplatform pilot analysis

**DOI:** 10.1017/cts.2017.227

**Published:** 2018-05-10

**Authors:** George Crowley, Sophia Kwon, Syed Hissam Haider, Liqun Zhang, Rachel Lam, Daniel Kim, Mengling Liu, David Prezant, Anna Nolan

**Affiliations:** NYU School of Medicine, New York, NY, USA

## Abstract

OBJECTIVES/SPECIFIC AIMS: In this pilot case-control study, the metabolome was quantified in subjects with previously measured serum and clinical biomarkers. The serum metabolome was then integrated with existing serum and clinical biomarkers of WTC-exposed firefighters to identify pathways significant to loss of lung function following acute PM-exposure. This robust subset of metabolite and serum biomarkers may be clinically relevant to predicting progression to lung disease in a larger cohort. METHODS/STUDY POPULATION: Serum drawn within 6 months of 9/11 was analyzed in this pilot. Clinical measures were obtained from electronic medical records. Never-smoking, male, WTC-exposed firefighters with normal pre-9/11 lung function were segregated based on FEV1 percent predicted (FEV1 %Pred) at symptomatic presentation. Cases of WTC-LI (FEV1 %Pred <LLN, n=15) and controls (n=15) were identified from previous cohorts. Ultrahigh performance liquid chromatography tandem mass spectroscopy quantified the metabolomic fingerprints of a group with previously assessed (by multiplex panels; ELISA and Luminex) serum chemokines and cytokines. High-dimensional data analysis and dimension reduction techniques integrated metabolites, cytokines, chemokines, and clinical data to identify pathways of WTC-LI on curated data. Random Forest (RF) out-of-bag estimated success rates were used to measure classification utility of the refined biomarker profile. Principal components analysis (PCA) was used to visualize class separation produced by the refined profile. RESULTS/ANTICIPATED RESULTS: Of the 765 metabolites detected, 580 metabolites were quantified in more than 80% of subjects/group with relative standard deviation ≥15%. Relevant chemokines, cytokines, and clinical biomarkers were included based on previously established clinical importance. Initial PCA explained 34.7% of the variance in the first 3 components. RF was used to identify the top 5% of biomarkers important to class separation. RF of the refined biomarker profile correctly classified cases and controls with a 96.7% estimated success rate. A PCA of the refined metabolic profile now explained 46.2% of the variance in components 1–3, demonstrating improved class separation. Differentiators between cases of WTC-LI and controls included elevated sphingolipids in cases of WTC-LI. The metabolic-inflammatory serum biomarkers MDC, Apo AI, GM-CSF, and heart rate play an important role in class separation. Phospholipids and lysolipids also appeared to differentiate cases of WTC-LI from controls. Specifically, several glycero-phosphatidylcholines (GPC) were elevated in cases of WTC-LI. DISCUSSION/SIGNIFICANCE OF IMPACT: High-dimensional data analysis on the metabolic fingerprints, serum, and clinical biomarker data of a subset of WTC-exposed 9/11 rescue workers has identified pathways associated with the loss of lung function. Sphingolipids, known to function as inflammatory signaling mediators, are thought to play important roles in lung function under both physiological and pathological conditions. Changes in sphingolipid metabolism have been linked to several pulmonary disorders, including asthma, COPD, and acute lung injury. Interestingly, a relation between sphingolipid metabolism and the metabolic-inflammatory pathway is suggested by similarities observed in PCA. Findings of elevated GPCs are similar to COPD literature. Higher levels of GPCs could correspond to elevated levels of lysophosphatidic acid (LPA), a ligand of RAGE. RAGE is a known proinflammatory mediator; LPA species have well-described roles as lipid signaling molecules, function as synthetic intermediates in other metabolic pathways, and were found to be predictive of WTC-LI. Since metabolites are more proximal markers of disease processes, metabolites could capture the complexity of past exposures and, therefore, may better inform treatment. These pathways warrant further investigation into their mechanisms and therapeutic importance.

